# Changes in biomarkers of the redox status in whole blood and red blood cell lysates in canine hypothyroidism

**DOI:** 10.1007/s11259-024-10382-4

**Published:** 2024-04-25

**Authors:** L. G. González-Arostegui, A. Muñoz-Prieto, G. García-López, J. J. Cerón, A. Tvarijonaviciute, C. P. Rubio

**Affiliations:** Interlab-UMU, Regional Campus of International Excellence “Mare Nostrum” University of Murcia, Murcia, 30100 Spain

**Keywords:** Antioxidants, Biomarkers, Dogs, Hypothyroidism, Oxidants, Whole blood

## Abstract

Hypothyroidism is the most commonly diagnosed endocrine disease in dogs. The objective of this study was to evaluate the changes in the redox status in canine hypothyroidism using whole blood (WB) and red blood cell (RBCs) lysates. For this purpose, a panel of five antioxidants and five oxidants biomarkers was measured in WB and RBCs lysates of 30 dogs with hypothyroidism, 26 dogs with non-thyroidal illnesses and 15 healthy dogs. The antioxidants measured were cupric reducing antioxidant capacity (CUPRAC), ferric reducing ability of plasma (FRAP), Trolox equivalent antioxidant capacity (TEAC), thiol and paraoxonase type-1 (PON-1). Oxidants measured include the total oxidant status (TOS), peroxide-activity (POX-Act), reactive oxygen-derived metabolites (d-ROMs), advanced oxidation protein products (AOPP) and thiobarbituric acid reactive substances (TBARS). WB showed a significant decrease of the antioxidants CUPRAC, TEAC and thiol, and also an increase in TBARS and a decrease in AOPP in dogs with hypothyroidism compared to healthy dogs. Meanwhile, RBCs lysates showed a significant increase in FRAP and PON-1 in dogs with hypothyroidism. The changes in the redox biomarkers in this study show that WB in canine hypothyroidism had a higher number of changes in biomarkers of the redox status than RBCs lysates, making it a promising sample type for the evaluation of the redox status in this disease. In addition, WB is easier and simpler to process than RBCs lysates and unlike serum, it does not have any hemolysis interference.

**Supplementary Information:**

The online version contains supplementary material available at 10.1007/s11259-024-10382-4.

## Introduction

Canine hypothyroidism is considered the most common endocrinopathy in dogs (Panciera 1994; Dixon and Reid 1999; Mooney 2011) and is characterized by an impairment of the thyroid gland, mainly produced by idiopathic atrophy or immune-mediated destruction of the gland (Feldman and Nelson 2015). This leads to a deficiency in the production of thyroid hormones, named triiodothyronine (T3) and thyroxine (T4), which as a consequence causes a decrease in the metabolic rate, leading to symptoms associated like weight gain, tiredness and intolerance to cold (Scott-Moncrieff 2007; Jaiswal et al. 2018).

Thyroid hormones can heavily influence the oxidative status (Chainy and Sahoo 2020). The effects of hypothyroidism on redox biomarkers have been reported thoroughly in humans. These show decreased antioxidants in serum, such as total antioxidant status (TAS), total thiol, paraoxonase type-1 (PON-1) (Azizi et al. 2003; Ates et al. 2015, 2016a; Al-Naimi et al. 2018), as well as, a decrease in plasmatic ferric reducing ability of plasma (FRAP) and superoxide dismutase (SOD) in red blood cells (RBCs) lysates (Reddy et al. 2013). Additionally, in RBCs lysates an increase in the antioxidant glutathione peroxidase (GPX) has been described, which could be a reflection of the high levels of circulating TSH and an increase in the antioxidant defenses (Reddy et al. 2013). On the other hand, hypothyroidism produces an increase in oxidant compounds in humans, with an increase found in plasma, serum, and RBCs lysates of thiobarbituric acid reactive substances (TBARS) due to lipid peroxidation (Torun et al. 2009; Reddy et al. 2013; Masullo et al. 2018). These changes have been shown to ameliorate when levothyroxine replacement therapy is used (Ates et al. 2016a; Al-Naimi et al. 2018; Masullo et al. 2018).

In canine hypothyroidism, an increase in serum oxidant biomarkers, namely the total oxidant status (TOS), peroxide-activity (POX-Act), reactive oxygen-derived compounds (d-ROMs) and TBARS were reported (Ryad et al. 2021; Arostegui et al. 2023). In addition, an increase in serum antioxidants such as Trolox equivalent antioxidant capacity (TEAC) and PON-1 were found (Arostegui et al. 2023). Although measurement of redox biomarkers is commonly performed in serum, whole blood (WB) and RBCs lysates can be used (González-Arostegui et al. 2022a). The use of samples different than serum, like WB and RBCs lysates offer new opportunities in the study of biomarkers of canine hypothyroidism or other diseases, leading to the development of new methods to evaluate the redox state that in the case of WB has the advantage of being an easier to process sample.

We hypothesized that biomarkers of the redox status could change in WB and RBCs lysates of dogs with hypothyroidism. Therefore, the objective of this study was to evaluate a panel of oxidants and antioxidants in both WB and RBCs lysates from dogs with hypothyroidism and compare them with dogs with non-thyroidal diseases and healthy dogs.

## Materials and methods

### Study design and case selection

Client-owned dogs attending several Veterinary Clinics of the Murcia Region (Spain), between March and October 2022, were included in this case-control study. Samples from a total of 71 dogs were used for the assessment of the redox status. This project has been approved by the Murcia University Ethics Committee with the number CEFA 288/2017.

The hypothyroidism group was integrated by dogs that meet the following inclusion criteria (González-Arostegui et al. 2022b): (1) being adult dogs (>1y), (2) absence of any other disease, (3) not having received any treatment six months before diagnosis, (4) clinical manifestation of the specific disease justifying the use of specific diagnostic tests (lethargy, tiredness, weight gain) (Mooney 2011) and (5) having specific test results indicating the presence of the disease (determination of T4 and TSH) (Lathan 2023). On the other hand, the non-hypothyroid diseased group was integrated by dogs with clinical signs compatible with hypothyroidism and specific test results indicating the absence of hypothyroidism. Lastly, a control group of healthy client-owned dogs that attended clinics for routine check-ups were included in this study after ruling out the presence of any disease. The nutritional status of all dogs was reported using the BCS 5-scale (1-thin; 2-underweight; 3-optimal (lean); 4-overweight; 5-obese) (Jagatheesan et al. 2017). Owner consent was obtained in all cases.

#### Sample preparation

Blood samples were obtained from jugular venipuncture and placed into EDTA and serum tubes. Serum tubes were left to clot at room temperature (22–24 °C) for 10 to 20 min, tubes were then centrifuged at 3500 g for 5 min at room temperature, the obtained serum was transferred to 1.5 mL Eppendorf® tubes and stored at -80 °C until analysis. WB and RBCs lysates were prepared following a previously described protocol (González-Arostegui et al. 2022a). Briefly, a volume of WB was transferred to 1.5 mL Eppendorf® tubes and stored at -80 °C until analysis, meanwhile, the remaining WB was centrifuged at 3000 rpm for 10 min at 4 °C in order to remove plasma and buffy coat. The RBCs pellet was then washed with isotonic saline (NaCl 0.9%) and centrifuged as previously mentioned. Then the supernatant was removed, and the process was repeated for a total of four washes, finally, the RBCs were lysed using ultrapure water in a 1:4 dilution and then stored at -80 °C until analysis.

### Analysis

The panel of redox biomarkers used in this study included five antioxidants, namely the cupric reducing antioxidant capacity (CUPRAC), FRAP, TEAC, thiol and PON-1, and five oxidants, namely TOS, POX-Act, d-ROMs, advanced oxidation protein products (AOPP) and TBARS. All the assays were performed in an automated biochemistry analyzer (Olympus AU400 Automatic Chemistry Analyzer, Olympus Europe GmbH, Germany), unless stated otherwise.

#### Antioxidant status

All the assays used to assess the antioxidant status of canine WB and RBCs lysates were previously validated (González-Arostegui et al. 2022a). The assays were done in the same way for all sample types.

CUPRAC assay was based on the reduction of Cu^2+^ into Cu^1+^ by the nonenzymatic antioxidants in the sample (Campos et al. 2009).

FRAP assay was based on the reduction of ferric-tripyridyltriazine (Fe^3+^-TPTZ) to the ferrous (Fe^2+^-TPTZ) form (Benzie and Strain 1996).

Measurement of TEAC was based on the assay described by Arnao et al. (Arnao et al. 1996) Its principle is based on the enzymatic generation of 2,2’-azino-bis(3-ethylbenz-thiazoline-6-sulfonic acid) (ABTS) radical and its reduction by non-enzymatic antioxidants present in the sample (Arnao et al. 1996).

The determination of thiol was based on the reaction of thiols within the sample with 5,5’-dithiobis-(2-nitrobenzoic acid) (DTNB)(Jocelyn 1987; Da Costa et al. 2006).

Measurement of PON-1 was based on the hydrolysis of phenyl acetate into phenol (Tvarijonaviciute et al. 2012).

#### Oxidant status

All the assays used to assess the oxidant status of canine WB and RBCs lysates were previously validated (González-Arostegui et al. 2022a). The assays were done in the same way for all sample types.

Determination of TOS was based on the assay described by Erel (Erel 2005). Its reaction is based on the ability of oxidants in the sample to oxidize Fe ^2+^-*o*-dianisidine complex to Fe^3+^ (Erel 2005).

POX-Act assay was based on the determination of total peroxides through a peroxide-peroxidase reaction using tetramethylbenzidine as the chromogenic substrate (Tatzber et al. 2003).

d-ROMs assay was based on the reaction of the sample in an acidic medium in the presence of *N*,*N*,-diethyl-*para*-phenylendiamine (DEPPD).

AOPP determination was based on oxidized albumin and di-tyrosine containing cross linked proteins, as previously described (Witko-Sarsat et al. 1996).

Determination of TBARS was based on the reaction of the sample to a Trichloroacetic acid, thiobarbituric acid and *N* hydrochloric acid stock (15% w/v trichloroacetic acid; 0.375% w/v thiobarbituric acid; 0.25 *N* hydrochloric acid) in heated conditions (Buege and Aust 1978). TBARS was measured using a microplate reader (Powerwave XS, Biotek Instruments).

### Statistical analysis

Data were analyzed using GraphPad Prism software (GraphPad Software Inc., version 9.3 for MacOS). The Shapiro-Wilk test was first used to assess whether data were normally distributed. Differences in the concentrations between groups were assessed by means of one-way ANOVA followed by Tukey’s range test when normally distributed and by means of one-way ANOVA followed by Kruskal-Wallis test when non-normally distributed. Correlation between serum and WB, serum and RBCs lysates and, WB and RBCs lysates were assessed using Pearson’s and Spearman’s correlation tests, according if the data were normally distributed or not. Statistical differences were considered for *p*-values < 0.05.

## Results

### Dog characteristics

A total of 71 dogs were included in this study. The hypothyroidism group (*n* = 30) was integrated by 25 males and 5 females, with a mean age of 8.9 years (range 6.0–13.0 years, SD 2.43), a mean BCS of 3.8/5 (range 2–5/5) and belonged to eleven different breeds being Beagle the most representative (*n* = 12). The non-hypothyroid diseased group (*n* = 26) was integrated by 17 males and 9 females, with a mean age of 8.6 years (range 4.0–14.0 years, SD 2.75), a mean BCS of 3.7/5 (range 2–5/5), being Mongrel (*n* = 7) the most common within the twelve different breeds of this group. The Control group (*n* = 15) was integrated by 13 males and 2 females, with a mean age of 6.7 years (range 4.0–7.0, SD 0.79), a mean BCS of 3.2/5 (range 2–4/5), the most common breed was Beagle (*n* = 13) among three different breeds. An individual description of the dogs involved in the study is presented in supplementary data (Supplementary Table S1). There were no statistically significant differences between groups in terms of age, BCS and gender.

### Redox status

#### Antioxidants status

**Fig. 1:**
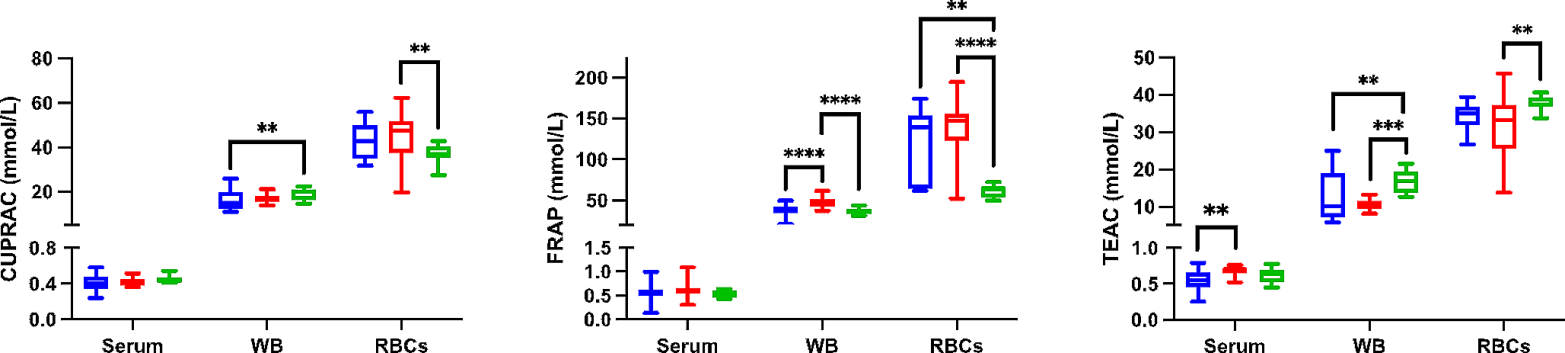
Results for antioxidant biomarkers for whole blood (WB) and red blood cells (RBCs) lysates. Cupric reducing antioxidant capacity (CUPRAC), ferric reducing ability of plasma (FRAP) and Trolox equivalent antioxidant capacity (TEAC) in dogs with hypothyroidism (blue box), non-thyroid diseases (red box) and controls (green box). Asterisks indicate significant differences between groups. **p ≤ 0.01, ***p ≤ 0.001, ****p ≤ 0.0001

Dogs with hypothyroidism showed lower levels of CUPRAC (*p* = 0.003), TEAC (*p* = 0.005) and thiol (*p ≤* 0.0001) compared to the Control group in WB (Fig. 1). Meanwhile, in RBCs lysates, higher levels of FRAP (*p* = 0.002) and PON1 (*p* < 0.05) were found. When data obtained from samples of dogs with hypothyroidism were compared with those of dogs with non-thyroidal diseases, lower FRAP values were observed in WB (*p ≤* 0.0001), while in serum lower TEAC (*p* = 0.002) was observed *p* (Fig. 1).

**Fig. 2:**
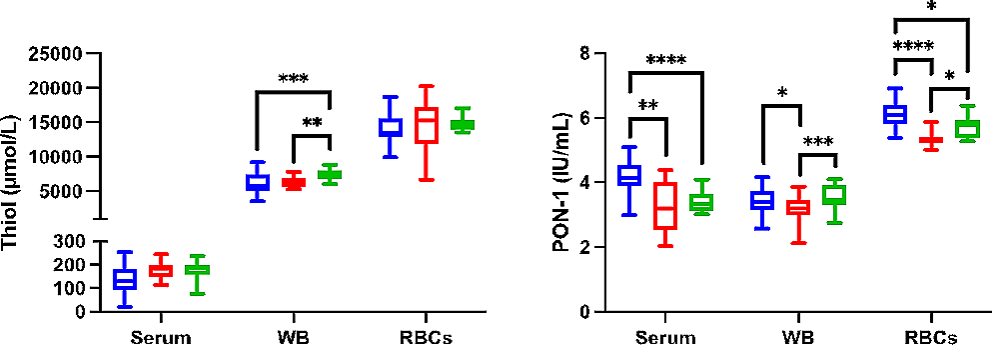
Results for antioxidant biomarkers for whole blood (WB) and red blood cells (RBCs) lysates. Thiol and paraoxonase type-1 (PON-1) in dogs with hypothyroidism (blue box), non-thyroid diseases (red box) and controls (green box). Asterisks indicate significant differences between groups. *p ≤ 0.05, **p ≤ 0.01, ***p ≤ 0.001, ****p ≤ 0.0001

On the other hand, WB, RBCs lysates and serum concentrations of PON-1 were higher in dogs with hypothyroidism compared to dogs with non-thyroidal diseases (*p =* 0.012, *p ≤* 0.0001, and *p* = 0.001, respectively) (Fig. 2).

#### Oxidant status

**Fig. 3:**
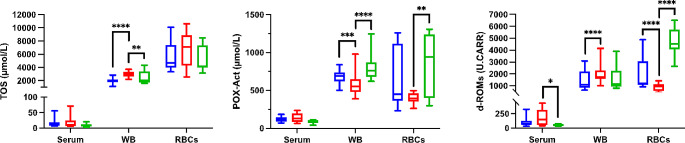
Results for oxidant biomarkers for whole blood (WB) and red blood cells (RBCs) lysates. Total oxidant status (TOS), peroxide-activity (POX-Act) and reactive oxygen-derived compounds (d-ROMs) in dogs with hypothyroidism (blue box), non-thyroid diseases (red box) and controls (green box). Asterisks indicate significant differences between groups. *p ≤ 0.05, **p ≤ 0.01, ***p ≤ 0.001, ****p ≤ 0.0001

**Fig. 4:**
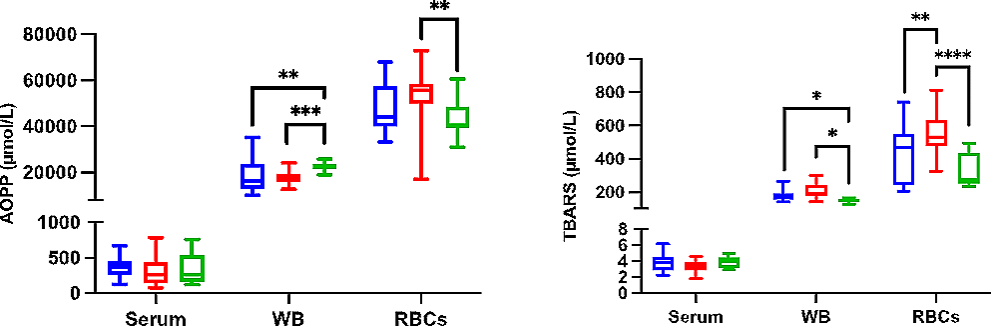
Results for oxidant biomarkers for whole blood (WB) and red blood cells (RBCs) lysates. Advanced oxidation protein products (AOPP) and thiobarbituric acid reactive substances (TBARS) in dogs with hypothyroidism (blue box), non-thyroid diseases (red box) and controls (green box). Asterisks indicate significant differences between groups. *p ≤ 0.05, **p ≤ 0.01, ***p ≤ 0.001, ****p ≤ 0.0001

In WB, TOS and d-ROMs concentrations were found to be lower in dogs with hypothyroidism compared to dogs with non-thyroidal diseases (*p ≤* 0.0001 and *p ≤* 0.0001, respectively), while POX-Act concentrations were found to be higher (*p =* 0.0005) (Fig. 3). When dogs with hypothyroidism were compared to the Control group, lower values of AOPP (*p* = 0.002) and higher values of TBARS (*p* = 0.034) were found in WB (Fig. 4). In RBCs lysates, concentrations of d-ROMs were found to be higher in dogs with hypothyroidism compared to dogs with non-thyroidal illnesses (*p ≤* 0.0001), while TBARS was found to be lower (*p =* 0.005). No statistically significant changes were observed in serum in the oxidant biomarkers between the different groups.

#### Correlation study

Correlations between results of serum and WB, serum and RBCs lysates, and WB and RBCs lysates are reported in Table 1. Between WB and RBCs lysates, the higher positive statistically significant correlations were found for FRAP and PON-1, and negative for AOPP and d-ROMs.

**Table 1:** Correlation coefficients (*r*) between serum and whole blood (WB), serum and red blood cells (RBCs) lysates, and WB and RBCs lysates

Correlations			
Biomarker	Serum VS WB (r)	Serum VS RBCs (r)	WB VS RBCs (r)
CUPRAC	-0.1576	0.3669 **	-0.1687
FRAP	-0.1832	-0.2724 *	0.4001***
TEAC	-0.3009 *	0.0034	0.1651
Thiol	-0.01294	0.2562	-0.1587
PON-1	0.1098	0.3228*	0.4131***
TOS	0.2561	0.1213	0.3529**
POX-Act	-0.1511	0.0076	0.0297
d-ROMs	0.1160	-0.2555	-0.7225****
AOPP	-0.3910 **	0.2238	-0.4553****
TBARS	-0.1145	-0.1986	0.4079

Cupric reducing antioxidant capacity (CUPRAC), ferric reducing ability of plasma (FRAP), Trolox equivalent antioxidant capacity (TEAC), paraoxonase type-1 (PON-1), total oxidant status (TOS), peroxide-activity (POX-Act), reactive oxygen-derived compounds (d-ROMs), advanced oxidation protein products (AOPP) and thiobarbituric acid reactive substances (TBARS). *p ≤ 0.05, **p ≤ 0.01, ***p ≤ 0.001, ****p ≤ 0.0001.

## Discussion

In this study, changes in different antioxidant and oxidant biomarkers were detected in WB and RBCs lysates of dogs with hypothyroidism indicating an altered redox status associated to this disease. To the author’s knowledge, this is the first report on the redox status biomarkers in WB and RBCs lysates in dogs with hypothyroidism.

In WB, a decrease in CUPRAC was found in dogs with hypothyroidism compared to the control group. We hypothesized that the decrease in CUPRAC concentrations could be associated with the oxidative stress state that occurs in hypothyroidism (Mancini et al. 2016; Kochman et al. 2021; Roshni et al. 2021). In addition, TEAC concentrations were lower in WB of dogs with hypothyroidism. Overall, the decreased CUPRAC and TEAC found in WB, could indicate a general decrease of the total antioxidant capacity as described in hypothyroidism in humans (Resch et al. 2002; Mancini et al. 2010). When individual antioxidants were evaluated, dogs with hypothyroidism showed decreased levels of thiol in WB when compared to the control group. This would be in line with reports found in humans indicating that serum thiol concentrations are decreased in patients with untreated Hashimoto’s thyroiditis (Ates et al. 2016b) and Graves’ disease (Agan et al. 2019) and would be in agreement with the decreases found in other antioxidants such as CUPRAC and TEAC in our study. Thiols play a critical role as part of the antioxidant defense system, protecting cells from oxidative stress as scavengers of hydrogen peroxide through protein signaling (Cremers and Jakob 2013; Ulrich and Jakob 2019). Overall the decrease in the antioxidants CUPRAC, TEAC, and thiol in our study could be associated with the lower amounts of thyroid hormones that are available to contribute to the synthesis of non-enzymatic antioxidants as it has been indicated in humans (Villanueva et al. 2013; Faam et al. 2021). This could be also the reason for the lower WB FRAP values found in dogs with hypothyroidism compared with dogs with other diseases in our study.

On the other hand, an increase in PON-1 was found in RBCs lysates of dogs with hypothyroidism compared to the control group. This increase is in agreement with a previous report of dogs with hypothyroidism (Arostegui et al. 2023). Interestingly, the results found in dogs are opposite to those found in humans, where a decrease in PON-1 is commonly associated with hypothyroidism (Azizi et al. 2003; Baskol et al. 2007; Ates et al. 2015; Al-Naimi et al. 2018). The difference found between dogs and humans is associated with the transport of PON-1 in high-density lipoproteins (HDL) in the latter, instead of low-density lipoproteins (LDL) as it happens in dogs (Downs et al. 1993; Rochu et al. 2010). This could be the reason for the higher PON-1 values found in RBCs lysates in this study since hypothyroidism induces dyslipidemia leading to the mobilization of LDLs (Pucci et al. 2000; Rizos et al. 2011).

In our study, when oxidants were studied, AOPP levels were lower and TBARS higher in WB of dogs with hypothyroidism compared to the Control group. Results found for AOPP in WB are in agreement with those found in saliva of dogs with hypothyroidism (Arostegui et al. 2023). Thyroid hormones promote protein metabolism, more specifically, albumin metabolism, therefore we hypothesize the lack of thyroid hormones could imply a decreased albumin metabolism resulting in a decreased oxidation of albumin, which is heavily associated with AOPP (Müller and Seitz 1984). In the same way, TOS and d-ROMs in WB were found to be decreased in hypothyroidism dogs when compared with those suffering from non-thyroid diseases which could be a consequence of thyroid dysfunction (Villanueva et al. 2013; Faam et al. 2021). On the other hand, the increase found for TBARS in WB was also found in RBCs lysates and serum of humans with hypothyroidism (Baskol et al. 2007; Erdamar et al. 2008; Öztürk et al. 2012; Santi et al. 2012; Masullo et al. 2018). The TBARS reaction serves as an indirect measurement of malondialdehyde (MDA), which is a product of lipid peroxidation (Gaweł et al. 2004). Due to the effects of hypothyroidism on lipid metabolism, this disease commonly causes dyslipidemia, which results in an increase of lipids and as a result an increased oxidation of these lipids (Pucci et al. 2000; Rizos et al. 2011). We hypothesized that the increase in TBARS found in WB from hypothyroid dogs could be associated with the increased lipid profile found in canine hypothyroidism due to the effects of lowered thyroid hormones on lipid metabolism.

The use of WB and RBCs lysates to evaluate redox biomarkers represents an interesting sample type, being easy to process, especially WB. Additionally, concentrations of many biomarkers in these sample types are higher than in serum and plasma (González-Arostegui et al. 2022a). However, data related to the use of WB to evaluate the redox state in different diseases is yet to be done, and at this moment, data is scarce. In this study, most of the changes for hypothyroid dogs and healthy controls were found using WB, which makes it a promising sample type for the measurement of redox biomarkers. The differences found between WB and RBCs lysates in our study could be due to the fact that in RBCs lysates there are not included oxidants or antioxidants from plasma, as it happens in WB (Koren et al. 2010). The results found in RBCs lysates were corrected by total hemoglobin concentration of each sample, however, no statistically significant differences were found among the corrected and uncorrected values (data not shown), as it has been described in a previous report (González-Arostegui et al. 2022a).

In this study, serum was evaluated to compare it with WB and RBCs lysates. Findings of these results indicate that serum had a significant correlation with two biomarkers in WB (TEAC and AOPP) and three biomarkers in RBCs lysates (CUPRAC, FRAP and PON-1). A statistically significant increase in PON-1 in hypothyroid dogs was found in serum being in agreement with a previous study in which this analyte was evaluated in serum in this disease (Arostegui et al. 2023), and this increase highlights the capability of PON-1 as a potential serum biomarker for canine hypothyroidism. Also in this report, TOS, POX-Act and d-ROMs concentrations showed a tendency to increase, while AOPP showed a tendency to decrease in dogs with hypothyroidism compared to healthy dogs, being also in line with a previous report, although in the present study the changes were not significant (Arostegui et al. 2023).

This study has some limitations. Most of the data was compared with data from humans since data about the use of these sample types for evaluation of redox status is scarce. However this comparison could be of interest specially in the case of hypothyroidism since dogs can be a model for this disease in humans (Bianchi et al. 2020). The results of other previous reports about the redox state in human hypothyroidism and those provided in this study were compared, and this comparison leads to some similarities, like a decreased antioxidant capacity and an increased lipid peroxidation (Resch et al. 2002; Ates et al. 2015; Chakrabarti et al. 2016). In addition this study should be considered a pilot study, as further research should be made using larger populations in order to corroborate these results and to evaluate the potential use of the measurement of these biomarkers in WB in clinical practice, where it would be a practical sample to use since it is easy to prepare and, unlike serum, it does not have the interference of hemolysis.

## Conclusions

WB showed changes in various redox biomarkers in canine hypothyroidism compared with healthy dogs. Namely in WB, there was a decrease in the antioxidants CUPRAC, TEAC, and thiol, and the oxidants AOPP and TBARS showed a decrease and an increase, respectively, in dogs with hypothyroidism. These changes would indicate a presence of oxidative stress in dogs with this disease and some of them were not detected in RBCs lysates and serum. Further studies should be made using larger populations in order to corroborate these results.

### Electronic supplementary material

Below is the link to the electronic supplementary material.

Supplementary Material 1

## Data Availability

No datasets were generated or analysed during the current study.
