# Supplementary material for: Changes in biomarkers of the redox status in whole blood and red blood cell lysates in canine hypothyroidism
Source: Vet Res Commun. 2024 Apr 25;48(4):2185–92. doi: 10.1007/s11259-024-10382-4 (PMC11315793; doi:10.1007/s11259-024-10382-4)
Supplement: Supplementary file 1 — Supplementary Material 1 [file 11259_2024_10382_MOESM1_ESM.docx]

| **Supplementary Table S1.** Individual characteristics of dogs included in this study. | | | | | |
| --- | --- | --- | --- | --- | --- |
| **Group** | **Dog** | **Gender** | **Breed** | **Age (years)** | **BCS (1-5)** |
| Hypothyroidism Group | 1 | Male | Labrador Retriever | 6 | 4 |
|  | 2 | Male | Golden Retriever | 9 | 4 |
|  | 3 | Male | Labrador Retriever | 8 | 4 |
|  | 4 | Male | German Shepherd | 8 | 3 |
|  | 5 | Female | Mongrel | 13 | 5 |
|  | 6 | Male | Hound | 11 | 4 |
|  | 7 | Male | Mongrel | 13 | 4 |
|  | 8 | Male | Mongrel | 7 | 3 |
|  | 9 | Female | Yorkshire Terrier | 13 | 4 |
|  | 10 | Female | Mongrel | 8 | 5 |
|  | 11 | Male | American Staffordshire | 9 | 4 |
|  | 12 | Female | Spanish Water dog | 6 | 4 |
|  | 13 | Male | Beagle | 7 | 4 |
|  | 14 | Male | Beagle | 7 | 3 |
|  | 15 | Male | Beagle | 7 | 3 |
|  | 16 | Male | Mongrel | 13 | 4 |
|  | 17 | Male | Swiss Shepherd | 11 | 4 |
|  | 18 | Male | Mongrel | 11 | 5 |
|  | 19 | Female | Mongrel | 13 | 4 |
|  | 20 | Male | Golden Retriever | 10 | 4 |
|  | 21 | Male | Boxer | 12 | 3 |
|  | 22 | Male | Beagle | 7 | 3 |
|  | 23 | Male | Beagle | 8 | 3 |
|  | 24 | Male | Beagle | 6 | 5 |
|  | 25 | Male | Beagle | 6 | 4 |
|  | 26 | Male | Beagle | 8 | 4 |
|  | 27 | Male | Beagle | 9 | 5 |
|  | 28 | Male | Beagle | 8 | 4 |
|  | 29 | Male | Beagle | 7 | 4 |
|  | 30 | Male | Beagle | 7 | 2 |
| Non-hypothyroid diseased Group | 1 | Female | Maltese Bichon | 9 | 4 |
|  | 2 | Male | English Bulldog | 7 | 3 |
|  | 3 | Male | Yorkshire Terrier | 9 | 5 |
|  | 4 | Female | Mongrel | 10 | 4 |
|  | 5 | Male | German Shepherd | 6 | 3 |
|  | 6 | Female | Mongrel | 10 | 5 |
|  | 7 | Male | Yorkshire Terrier | 8 | 4 |
|  | 8 | Male | Hound | 5 | 2 |
|  | 9 | Male | Boxer | 12 | 3 |
|  | 10 | Female | Yorkshire Terrier | 13 | 4 |
|  | 11 | Female | Mongrel | 12 | 2 |
|  | 12 | Male | Mongrel | 9 | 4 |
|  | 13 | Male | Mongrel | 14 | 3 |
|  | 14 | Male | Mongrel | 14 | 3 |
|  | 15 | Female | Mongrel | 12 | 4 |
|  | 16 | Male | Shar pei | 6 | 5 |
|  | 17 | Female | Labrador Retriever | 4 | 4 |
|  | 18 | Male | Golden Retriever | 8 | 5 |
|  | 19 | Female | Yorkshire Terrier | 8 | 4 |
|  | 20 | Female | Pomeranian | 6 | 4 |
|  | 21 | Male | Beagle | 7 | 4 |
|  | 22 | Male | Beagle | 7 | 3 |
|  | 23 | Male | Beagle | 7 | 5 |
|  | 24 | Male | Beagle | 7 | 5 |
|  | 25 | Male | Beagle | 7 | 2 |
|  | 26 | Male | Beagle | 7 | 4 |
| Control Group | 1 | Male | Beagle | 7 | 4 |
|  | 2 | Male | Beagle | 7 | 3 |
|  | 3 | Male | Beagle | 7 | 3 |
|  | 4 | Male | Beagle | 7 | 2 |
|  | 5 | Male | Beagle | 7 | 3 |
|  | 6 | Male | Beagle | 7 | 3 |
|  | 7 | Male | Beagle | 7 | 4 |
|  | 8 | Male | Beagle | 7 | 4 |
|  | 9 | Male | Beagle | 7 | 3 |
|  | 10 | Male | Beagle | 7 | 4 |
|  | 11 | Male | Beagle | 7 | 3 |
|  | 12 | Male | Beagle | 7 | 3 |
|  | 13 | Male | Beagle | 7 | 3 |
|  | 14 | Female | Yorkshire Terrier | 6 | 3 |
|  | 15 | Female | Malinois | 4 | 3 |
